# Necroptosis pathway emerged as potential diagnosis markers in spinal cord injury

**DOI:** 10.1111/jcmm.18219

**Published:** 2024-03-20

**Authors:** Jingcheng Liu, Jiang Cao, Xiao Yu, Jie Chang, Tao Sui, Xiaojian Cao

**Affiliations:** ^1^ Department of Orthopedics The First Affiliated Hospital with Nanjing Medical University Nanjing Jiangsu China; ^2^ Department of Orthopedics The Affiliated Hospital of Nanjing University Medical School Nanjing Jiangsu China

**Keywords:** bioinformatics, DEGs, GEO database, necroptosis, spinal cord injury

## Abstract

The present research focused on identifying necroptosis‐related differentially expressed genes (NRDEGs) in spinal cord injury (SCI) to highlight potential therapeutic and prognostic target genes in clinical SCI. Three SCI‐related datasets were downloaded, including GSE151371, GSE5296 and GSE47681. MSigDB and KEGG datasets were searched for necroptosis‐related genes (NRGs). Differentially expressed genes (DEGs) and NRGs were intersected to obtain NRDEGs. The MCC algorithm was employed to select the first 10 genes as hub genes. A protein–protein interaction (PPI) network related to NRDEGs was developed utilizing STRING. Several databases were searched to predict interactions between hub genes and miRNAs, transcription factors, potential drugs, and small molecules. Immunoassays were performed to identify DEGs using CIBERSORTx. Additionally, qRT‐PCR was carried out to verify NRDEGs in an animal model of SCI. Combined analysis of all datasets identified 15 co‐expressed DEGs and NRGs. GO and KEGG pathway analyses highlighted DEGs mostly belonged to pathways associated with necroptosis and apoptosis. Hub gene expression analysis showed high accuracy in SCI diagnosis was associated with the expression of *CHMP7 and FADD*. A total of two hub genes, i.e. *CHMP7, FADD,* were considered potential targets for SCI therapy.

## 1 INTRODUCTION

Spinal cord injury (SCI) constitutes an extremely debilitating and potentially lethal traumatic disease affecting the central nervous system (CNS). Severe SCI induces paraplegia, sensory loss and other functional abnormalities in the injured segment. These conditions impose an important financial burden on the affected individuals, the respective families and society. Excitotoxicity, neuroinflammation, lipid peroxidation, ischemia–reperfusion injury, and free radical production are key pathological processes and microenvironment imbalances following SCI that impede its repair.^1^, ^2^ SCI generally results in sensorimotor and autonomic nerve injuries and is still considered an important public health concern globally. About 273,000 Americans are affected by SCI, with an estimated 12,000 new cases reported annually.^3^ At present, reliable therapeutic strategies for SCI are lacking. Therefore, further in‐depth studies examining the underlying mechanisms are needed to improve the treatment in SCI.

Cellular death can be resulted from the cytotoxic effects of exogenous or endogenous compounds.^4^ Recently published reports have identified various modes of programmed cell death (PCD), e.g. autophagy, pyroptosis and necroptosis.^5^, ^6^, ^7^, ^8^ Necroptosis is involved in the development of various immune system‐related disorders such as cerebral ischemia and acute/chronic neurodegenerative disorders.^9^ Previous evidence reveals necroptosis is associated with inflammation post‐SCI.^10^, ^11^ Necroptosis is widely acknowledged as an important type of PCD after SCI. Necroptosis activation is associated with cell death and tissue damage in CNS injuries. Despite extensive research on the molecular mechanisms underlying SCI‐induced necroptosis, this process is not completely understood. Therefore, given the substantial involvement of necroptosis in SCI pathogenesis, identifying the molecular pathways underlying SCI‐induced necroptosis may open up the pool of genes for potential therapeutic targets.

In this work, three datasets for SCI (GSE151371, GSE5296 and GSE47681) were examined, and 159 necroptosis‐related differentially expressed genes (NRDEGs) were obtained from the Gene Expression Omnibus (GEO) and Kyoto Encyclopedia of Genes and Genomes (KEGG) databases. Necroptosis‐related genes (NRGs) were filtered utilizing the Molecular Signatures Database (MSigDB). Afterward, GO term and KEGG pathway analyses and protein–protein interaction (PPI) building were carried out to determine hub genes. Additionally, miRNA‐mRNA, TF‐mRNA and drug‐mRNA interaction networks were constructed. Immune cell infiltration was determined using CIBERSORTx. Finally, NRDEGs were verified by qRT‐PCR in an animal model of SCI. This work aimed to assess the molecular mechanisms of SCI‐induced necroptosis.

## 2 MATERIALS AND METHODS

### 2.1 Source of the gene datasets, data collection and correction

SCI‐associated RNA sequencing datasets, including GSE151371,^12^ GSE5296 and GSE47681^13^ were retrieved from GEO using the GEO query^14^ package. The GSE151371 dataset was obtained for *Homo sapiens* using the GPL20301 data platform. Of the 58 peripheral white blood cell (PWBC) specimens in the latter dataset, 10 were obtained from patients without a history of CNS pathology (normal), 10 were from individuals with non‐CNS trauma, and 38 were from patients with traumatic SCI. The analysis included the normal and SCI samples. The GSE5296 dataset was obtained from *Mus musculus* using the GPL1261 data platform. This dataset includes 96 spinal cord samples. A total of 18 traumatic SCI and 12 pseudo‐injury (Sham) specimens were included in the analysis. The GSE47681 dataset for *Mus musculus* using the GPL1261 data platform contained 34 spinal cord samples (Table S1). Additionally, 13 SCI samples with the trkB.T1 WT genotype at the trauma site and four Sham samples were included in the analysis. GSE151371 served as the test set, and GSE5296 and GSE47681 were combined to generate a validation set for subsequent gene expression validation.

To investigate the involvement of necroptosis in SCI, 159 NRGs were retrieved from the KEGG database,^15^ and eight NRGs specifically associated with the GOBP_NECROPTOTIC_SIGNALLING_PATHWAY were retrieved from MSigDB.^16^ These eight NRGs were combined with 159 NRGs from the KEGG database, resulting in a total of 159 NRGs after removing duplicates (Table S2).

‘sva’ in R was utilized to eliminate batch effects in the SCI datasets GSE5296 and GSE47681 to generate the merged GEO dataset. The datasets were then compared pre‐ and post‐removal of batch effects. Distribution box line (Figure S1A,B) and PCA (Figure S1C,D) plot analyses demonstrated a significant reduction in batch effect within the SCI dataset following the batch removal process. The GSE151371 dataset was corrected using ‘limma’ (Figure S1E,F), resulting in a significant reduction of the inter‐sample batch effect.

### 2.2 NRDEGs

To identify differentially expressed genes (DEGs) between different subgroups (SCI and normal), data correction on the GSE151371 dataset was carried out using the R package ‘limma.’ Subsequently, ‘sva’ in R^17^ was utilized to eliminate batch effects from GSE5296 and GSE47681, generating a combined dataset for subsequent validation. This combined dataset comprised 31 SCI and 16 Sham samples. Principal Component Analysis (PCA) was carried out to assess the dataset prior to and following the de‐batching process.^18^

‘limma’ in R was employed to assess the GSE151371 dataset for DEGs (|logFC| >1 and *p*.adjust <0.05). In this study, logFC >1 and logFC <−1 were considered to be upregulated and downregulated, respectively.

Differential analysis of the GSE151371 dataset was conducted to identify necroptosis‐related DEGs (|logFC| >1 and *p*.adjust <0.05) which were intersected with NRGs and Venn diagram analysis was carried out to identify NRDEGs. Visualization of data used volcano plots created with ‘ggplot2’ in R. Heat maps showing the NRDEGs were generated with ‘pheatmap’ in R.

### 2.3 GO and KEGG analyses of NRDEGs

GO analysis is widely used for conducting comprehensive functional enrichment analyses encompassing biological processes (BP), molecular functions (MF), and cellular components (CC).^19^ The KEGG database is extensively utilized to store genomic data, biological pathways, disease information, and drug‐related data. ‘ClusterProfiler’ in R^20^ was employed to annotate NRDEGs for GO terms (*p*.adjust <0.05 and FDR value [*q*.value] <0.20). The Benjamini‐Hochberg (BH) procedure was used for the correction of p‐values. Visualization of KEGG analysis results utilized ‘Pathview’ in R^21^ and displayed the associated pathway maps.

### 2.4 GSVA enrichment analysis

Gene set variation analysis (GSVA)^22^ was employed to assess the disparities of the enrichment of gene sets in functional pathways between the SCI and normal groups for the human samples from GSE151371 dataset. This is accomplished via transformation of the gene expression matrix between specimens into gene sets across samples. This method assesses pathway enrichment across multiple samples. The ‘h.all.v7.4.symbols.gmt’ gene set was acquired from MSigDB^16^ for GSVA of combined dataset samples. Afterward, functional disparities in the enriched pathways between the SCI and normal groups were assessed. The condition for significant enrichment was *p*.adjust <0.05.

### 2.5 Protein–protein interactions

PPI networks comprise proteins that engage in interactions with each other. STRING^23^ enables the search for known proteins and the prediction of their interactions. In the present work, STRING was employed to construct PPI networks for NRDEGs by setting biological species as humans with a minimum correlation coefficient >0.400. Cytoscape was employed to visualize the resulting PPI network models.^24^ In addition, the Maximal Clique Centrality (MCC) algorithm in cytoHubba^25^ was utilized to compute scores and the top 10 genes were considered hub genes.

### 2.6 mRNA‐miRNA, mRNA‐TF, and mRNA‐drug interaction network building

ENCORI (https://starbase.sysu.edu.cn/) version 3.0,^26^ an upgraded version of starBase, utilizes CLIP‐seq and degradome sequencing (for plants) data analysis to study a wide range of interactions such as miRNA‐ncRNA, ncRNA–RNA, miRNA‐mRNA, RBP‐ncRNA, RNA–RNA and RBP‐mRNA interactions. miRNA target gene prediction and functional annotation were carried out by searching the miRDB database.^27^ The ENCORI and miRDB databases were employed for predicting miRNAs that interact with hub genes and a mRNA‐miRNA interaction network was plotted by combining data from the miRDB database (Target Score >50) and mRNA‐miRNA data from ENCORI.

The CHIPBase 3.0 database (https://rna.sysu.edu.cn/chipbase/)^28^ contains multiple binding motif matrices and associated binding sites derived from the ChIP‐seq data of DNA‐binding proteins. Multiple transcription factors (TFs) and genes could be analysed to predict their modulatory relationships. The hTFtarget database (http://bioinfo.life.hust.edu.cn/hTFtarget)^29^ provides extensive information on human TFs and the related target genes. CHIPBase and hTFtarget were searched for the identification of TFs interacting with hub genes.

In addition, the Comparative Toxicogenomics Database (CTD)^30^ (http://ctdbase.org/) was utilized to identify drugs or small molecules potentially interacting with hub genes by selecting interactions that have been reported in three or more publications. Afterward, Cytoscape was utilized to depict the mRNA‐drug interaction network.

### 2.7 Immuno‐infiltration analysis using CIBERSORTx

GSE151371 gene expression matrix data uploaded to CIBERSORTx (https://cibersortx.stanford.edu/) platform^31^ were merged with the LM22 eigengene matrix for the generation of an immune cell‐infiltration matrix. Data showing immune cell enrichment scores above 0 were selected to yield the required outcomes for the immune cell infiltration matrix. ‘ggplot2’ in R was employed to generate histograms illustrating the contents of 22 infiltrated immune cell types in every specimen, and the R project ‘pheatmap’ was utilized to generate correlation heat maps. In addition, the associations of immune cells with hub genes were assessed using the ‘psych’ package and visualized using a correlation heat map.

### 2.8 Animal model

Animal experiments were conducted with female C57BL/6 mice housed under a 12‐h photoperiod at 25°C with freely available food and water.

Eight to twelve‐week‐old C57BL/6 mice (20–25 g) were analysed to ensure consistency in spinal cord size. To minimize the effect of varying levels of surgical expertise, the same surgeon performed all the SCI procedures. The animals were assigned to two groups randomly (*n* = 3/group): (1) Negative Control (NC) group, mice were exposed only to the spinal cord without any other special treatment; (2) SCI group, mice administered 2% pentobarbital (30 mg/kg) to induce anaesthesia, after which a 2‐cm incision was made along the dorsal midline. A T8 laminectomy was performed, followed by a 15‐s lateral compression of the spinal cord using specialized forceps at 0.4‐mm intervals, resulting in a precise moderate crush injury. The animals were subsequently returned to their cages upon regaining full consciousness. SCI was successfully modelled in mice, and spinal cord samples were collected 3 days post‐surgery for future use.

### 2.9 qRT‐PCR

Total RNA extraction from spinal tissue specimens utilized TRIzol reagent (Yifeixue Biotech, Nanjing, China), as directed by the manufacturer. Then, total RNA (1 μg) underwent reverse transcription utilizing HiScript III RT SuperMix for qPCR (+gDNA wiper) reverse transcriptase (Vazyme, China). qRT‐PCR was conducted on a Step‐One Plus Real‐Time PCR System (Applied Biosystems, USA) in 10‐μL reactions encompassing 100 ng cDNA, 200 nM of each primer, and 5 μL AceQ qPCR SYBR Green Master Mix (Vazyme). The expression of target genes was assessed by the 2^−ΔΔCT^ method, using GAPDH for normalization. The primers utilized are shown in Table S3.

### 2.10 Statistical analysis

R 4.2.0 and GraphPad Prism 9.0 were employed for data analysis. Independent samples Student's *t*‐test was conducted to determine group pair differences for continuous variates showing normal distribution. Comparisons of continuous data with skewed distribution in two and multiple groups utilized the Mann–Whitney *U*‐test (rank‐sum test) and the Kruskal‐Wallis test, respectively. For comparing categorical variates between two groups, the chi‐square test or Fisher's exact test was applied. Spearman's correlation analysis was utilized to calculate correlation coefficients for various parameters. Two‐tailed *p* < 0.05 indicated statistical significance unless otherwise stated.

## 3 RESULTS

### 3.1 Analysis of NRDEGs

**FIGURE 1:**
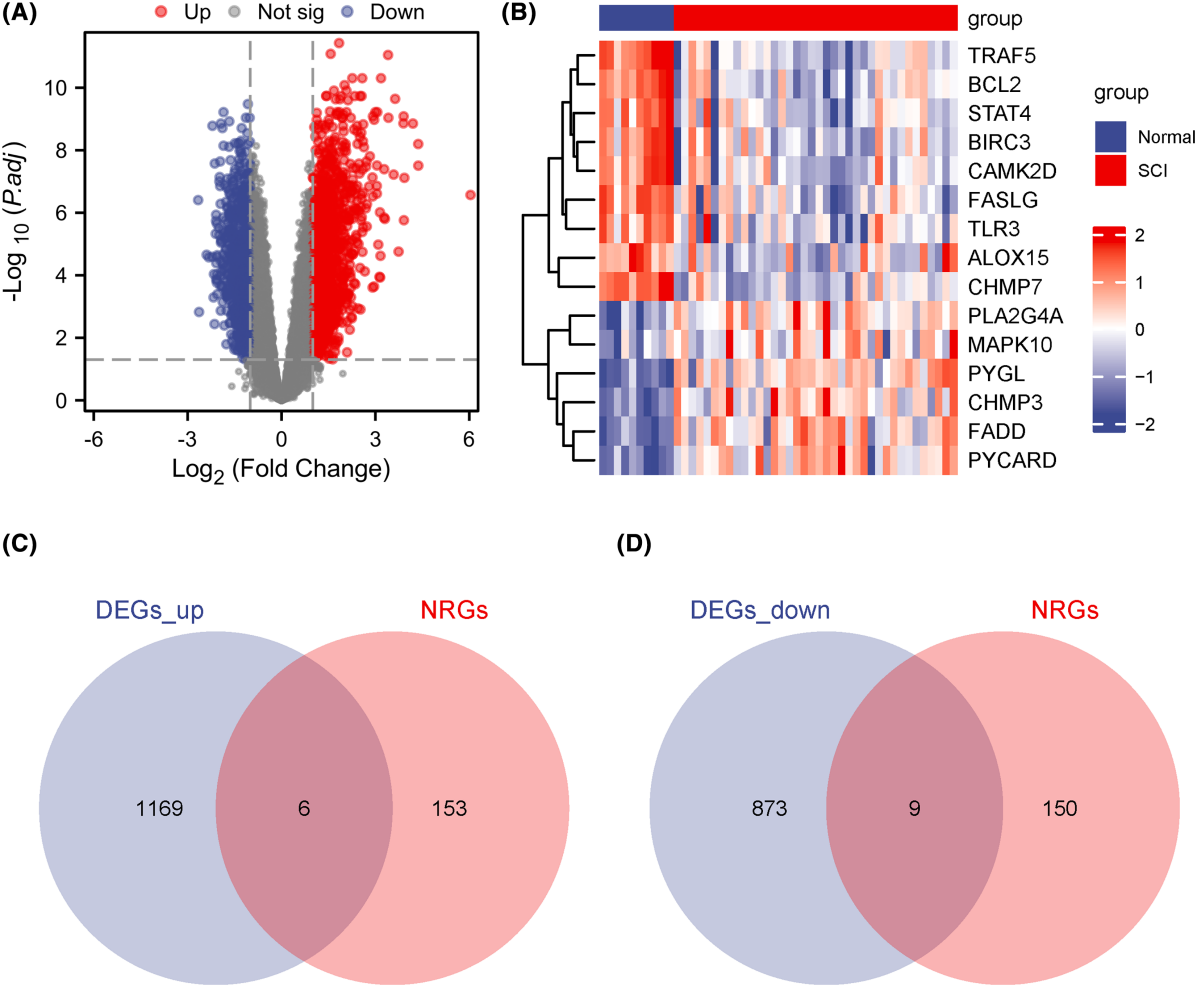
Differentially expressed genes (DEGs) related to necroptosis. (A) Volcano plot depicting DEGs in the spinal cord injury (SCI) and normal groups based on the merged dataset. (B) Heat map showing NRDEGs in the SCI and normal groups based on the combined dataset. Venn diagrams of upregulated (C) and downregulated (D) DEGs and NRGs are shown.

The GSE151371 dataset was subdivided into the SCI and control (normal) groups, which were compared with ‘limma’ in R for gene expression values. The technical approaches used for this analysis are shown in Figure S2. Interestingly, the analysis identified 2057 DEGs in the combined dataset (|logFC| >1 and *p*.adjust <0.05), with 1175 upregulated and 882 downregulated genes, which are shown in a volcano plot (Figure 1A). To identify NRDEGs, the intersection of DEGs and NRDEGs was analysed with logFC >1 and *p*.adjust < 0.05 (Figure 1C); as a result, six NRDEGs (*CHMP3, FADD, MAPK10, PLA2G4A, PYGL* and *PYCARD*) were identified. Additionally, by intersecting the obtained DEGs and NRDEGs with logFC<−1 and p.adjust <0.05 (Figure 1D), nine NRDEGs were identified (*ALOX15, BCL2, BIRC3, CHMP7, CAMK2D, FASLG, STAT4, TRAF5* and *TLR3*). Based on the intersection results, expression differences for NRDEGs between distinct subgroups in the joint dataset were assessed. Heat maps were generated to visualize these differences (Figure 1B).

### 3.2 Functional enrichment and pathway enrichment analyses of NRDEGs

**FIGURE 2:**
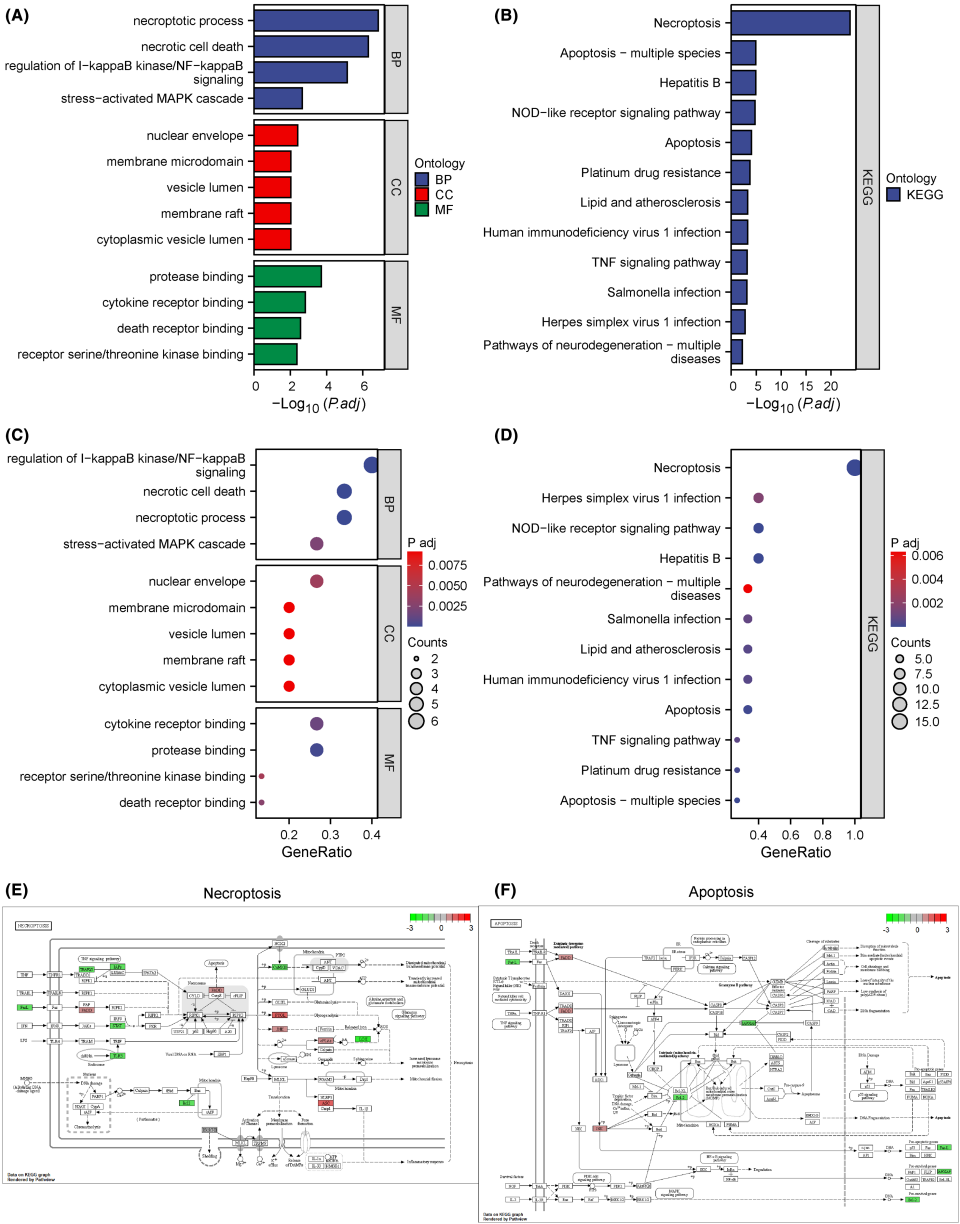
Function (GO) and pathway (KEGG) enrichment analyses of NRDEGs. (A) Histogram depicting GO analysis results for NRDEGs. (B) Histogram depicting KEGG pathway analysis results for NRDEGs. (C) Bubble plot showing GO function enrichment analysis results for NRDEGs. (D) Bubble plot depicting KEGG pathway analysis results for NRDEGs. (E) Necroptosis pathway. (F) Apoptosis pathway. In (E) and (F), colours correspond to the logFC in the differential analysis of NRDEGs. KEGG, Kyoto Encyclopedia of Genes and Genomes; NRDEGs, necroptosis‐related differentially expressed genes.

A total of 15 NRDEGs (*ALOX15, BCL2, BIRC3, CAMK2D, CHMP3, CHMP7, FASLG, FADD, MAPK10, PLA2G4A, PYCARD, PYGL, STAT4, TRAF5* and *TLR3*) were assessed for BP, MF, CC, and associated biological pathways. Moreover, GO analysis (Figure 2A) and KEGG pathway analysis (Figure 2B) of the NRDEGs were carried out. The association between NRDEGs and the outcomes of both analyses is also illustrated using bubble plots (Figure 2C,D).

As shown in Figure 2A–D, NRDEGs were primarily involved in the regulation of I‐kappaB kinase/NF‐kappaB signalling, necroptotic process, necrotic cell death, stress‐activated MAPK cascade, and other BP. These NRDEGs were also enriched in several CC, including cytoplasmic vesicle lumen, nuclear envelope, vesicle lumen, membrane raft and membrane microdomain. Furthermore, they were enriched in various MF such as protease binding, cytokine receptor binding, death receptor binding and receptor serine/threonine kinase binding (Table S4). The critical pathways involved in the enrichment of NRDEGs were necroptosis, Hepatitis B, NOD‐like receptor signalling pathway, apoptosis, HIV 1 infection, lipid and atherosclerosis, Salmonella infection, Herpes simplex virus 1 infection, pathways of neurodegeneration‐multiple diseases, and apoptosis‐multiple species. Additionally, pathways related to platinum drug resistance and TNF signalling were investigated (Table S5). Pathway maps for Necroptosis pathway (E) and Apoptosis pathway (F) are shown (Figure 2E,F).

### 3.3 GSVA data

**FIGURE 3:**
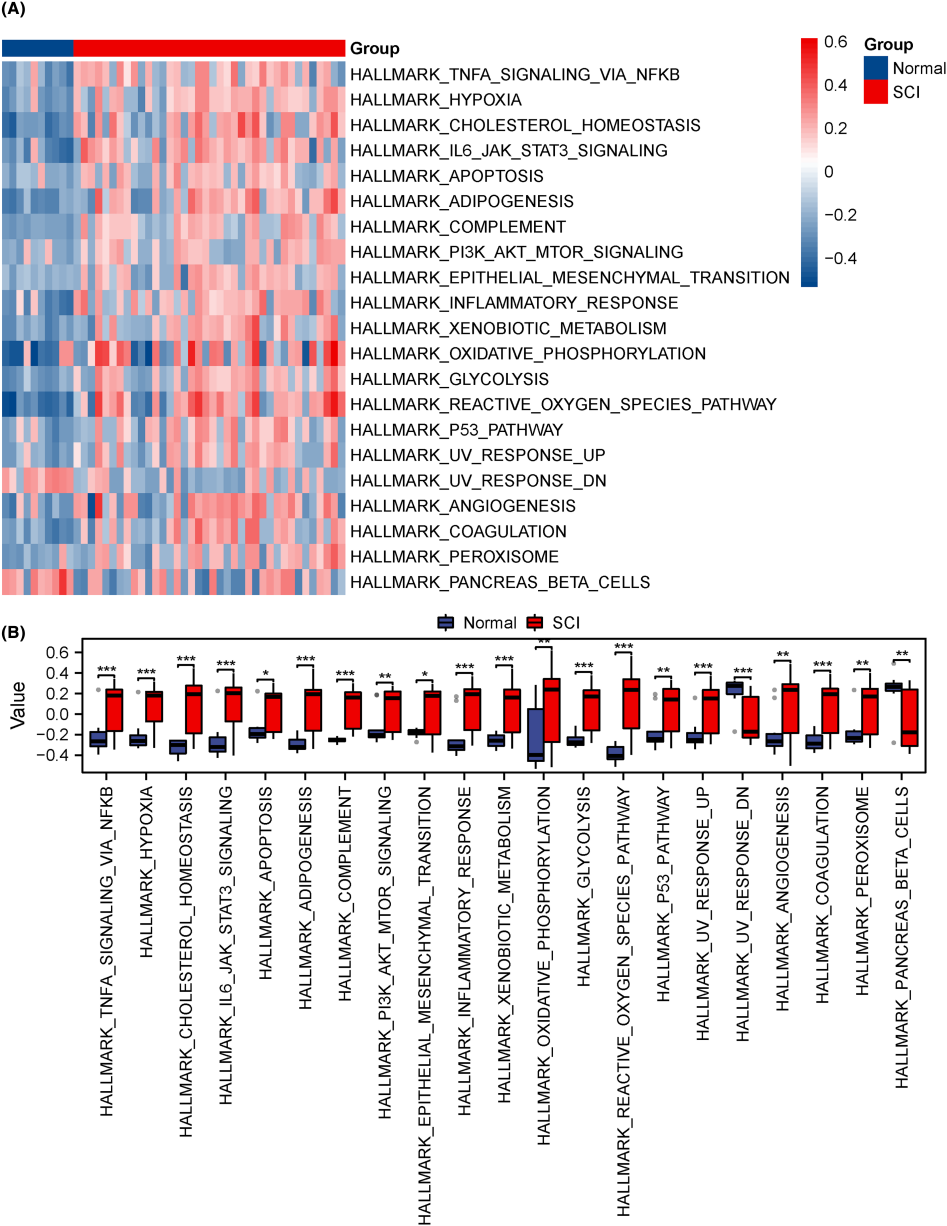
GSVA of the GSE151371 dataset. (A, B) Complex numerical heat map of GSVA enrichment analysis data for the GSE151371 dataset (A), grouped comparison plot (B). *p < 0.05; **p < 0.01; ****p < 0.0001. ns, not significant.

GSVA of the GSE151371 dataset was conducted to examine gene expression in the HALLMARK gene set between the SCI and normal groups (Table S6). The differential expression of 21 HALLMARK pathways was analysed among the different subgroups based on results obtained from GSVA. ‘pheatmap’ in R was employed to generate a heat map illustrating specific variation analysis (Figure 3A). Moreover, variations in the grouping of 21 HALLMARK pathways were assessed across different groupings of the TCGA‐LUAD dataset using a grouping comparison map (Figure 3B). In the GSE151371 dataset, 21 HALLMARK gene sets (HALLMARK_IL6_JAK_STAT3_SIGNALLING, HALLMARK_ADIPOGENESIS, HALLMARK_HYPOXIA, HALLMARK_GLYCOLYSIS, etc.) showed statistical significance (*p* < 0.05) between the SCI and normal groups.

### 3.4 Protein–protein interaction networks

PPI analysis considering 15 NRDEGs (*ALOX15, BCL2, BIRC3, CAMK2D, CHMP3, CHMP7, FASLG, FADD, MAPK10, PLA2G4A, PYCARD, PYGL, STAT4, TRAF5* and *TLR3*) was conducted with the aid of STRING (Figure S3A). A PPI network was constructed for the selected NRDEGs and the resulting interactions were observed using Cytoscape (Figure S3B). Among these NRDEGs, 12 (*ALOX15, BCL2, BIRC3, CHMP3, CHMP7, FASLG, FADD, MAPK10, PLA2G4A, STAT4, TRAF5* and *TLR3*) interacted with the other genes. The cytoHubba plugin was employed to assess the scores of NRDEGs using the MCC (Figure S3C) algorithm and selected the top 10 genes as hub genes (*ALOX15, BIRC3, CHMP3, CHMP7, FASLG, FADD, PLA2G4A, STAT4, TRAF5* and *TLR3*).

### 3.5 mRNA‐miRNA, mRNA‐TF, and mRNA‐drug interaction networks

**FIGURE 4:**
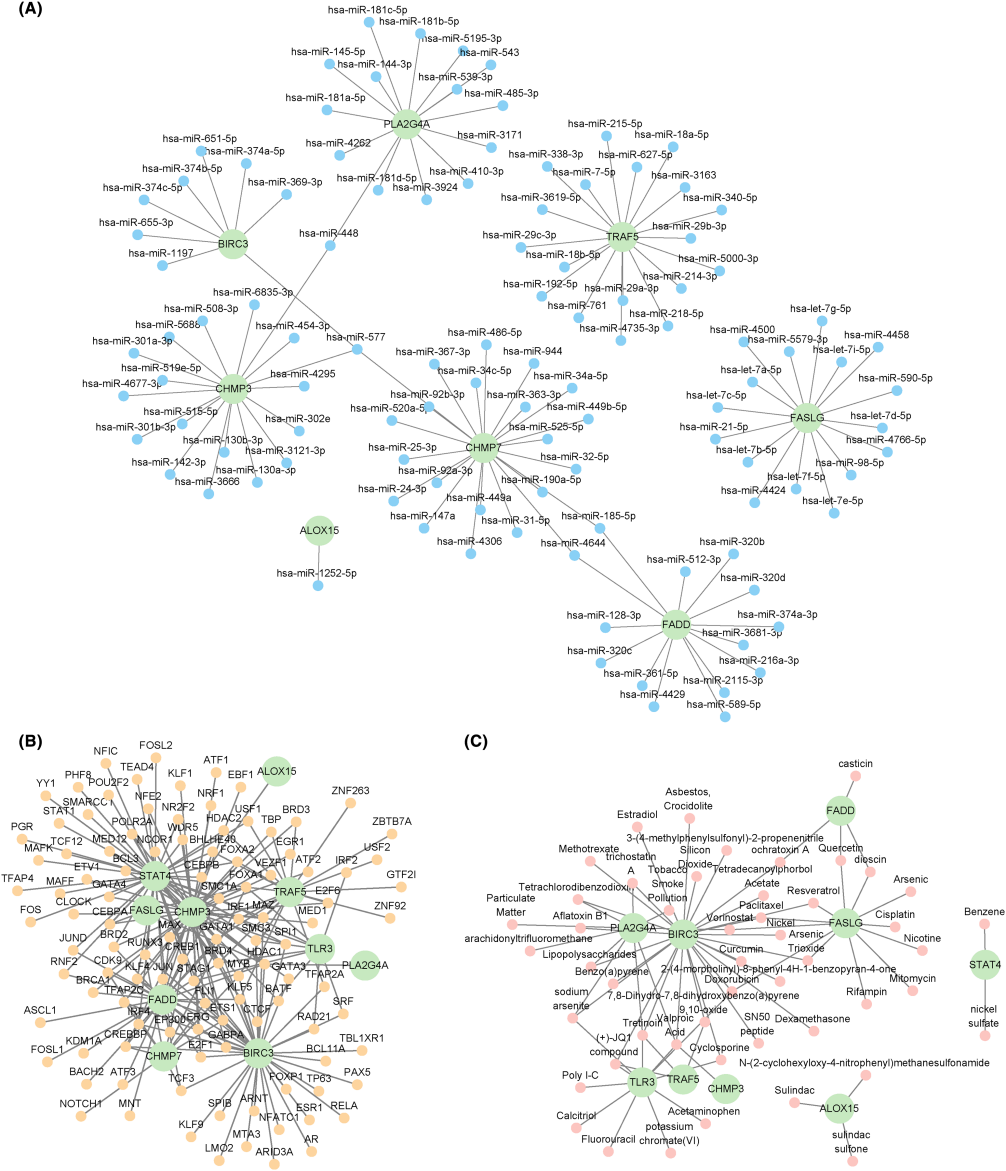
mRNA‐miRNA, mRNA‐TF, and mRNA‐drug interaction networks. (A–C) Hub genes of mRNA‐miRNA (A), mRNA‐TF (B) and mRNA‐drug (C) interaction networks. Green, blue, yellow and pink circular blocks denote mRNAs, miRNAs, TFs, and drugs, respectively.

mRNA‐miRNA data from the ENCORI and miRDB databases were employed for the prediction of the interactions of miRNAs with 10 hub genes (*ALOX15, BIRC3, CHMP3, CHMP7, FASLG, FADD, PLA2G4A, STAT4, TRAF5* and *TLR3*). These interactions were visualized with the Cytoscape (Figure 4A). The mRNA‐miRNA interaction network comprised eight hub genes (*ALOX15, BIRC3, CHMP3, CHMP7, FASLG, FADD, PLA2G4A* and *TRAF5*) and 107 miRNAs, resulting in totally 112 pairs of mRNA‐miRNA (Table S7).

TFs binding to hub genes were detected using the CHIPBase and hTFtarget databases. The interactions based on both databases were downloaded and intersected, resulting in the identification of 10 hub genes (*ALOX15, BIRC3, CHMP3, CHMP7, FASLG, FADD, PLA2G4A, STAT4, TRAF5* and *TLR3*) and 107 TFs (Figure 4B). The interaction relationship data were visualized with Cytoscape. The mRNA‐TF interaction network had the most interactions between the hub gene *CHMP3* and TFs. Notably, the *CHMP3* gene targeted 49 TFs simultaneously (Table S8).

The CTD database was employed for the identification of 10 hub genes with the potential for drug or molecular compound development. In the mRNA‐drug interaction network (Figure 4C), 49 potential drugs or molecular compounds were associated with nine hub genes (*ALOX15, BIRC3, CHMP3, FASLG, FADD, PLA2G4A, STAT4, TRAF5* and *TLR3*) (Table S9).

### 3.6 Immuno‐infiltration analysis of the GSE151371 dataset using CIBERSORTx

**FIGURE 5:**
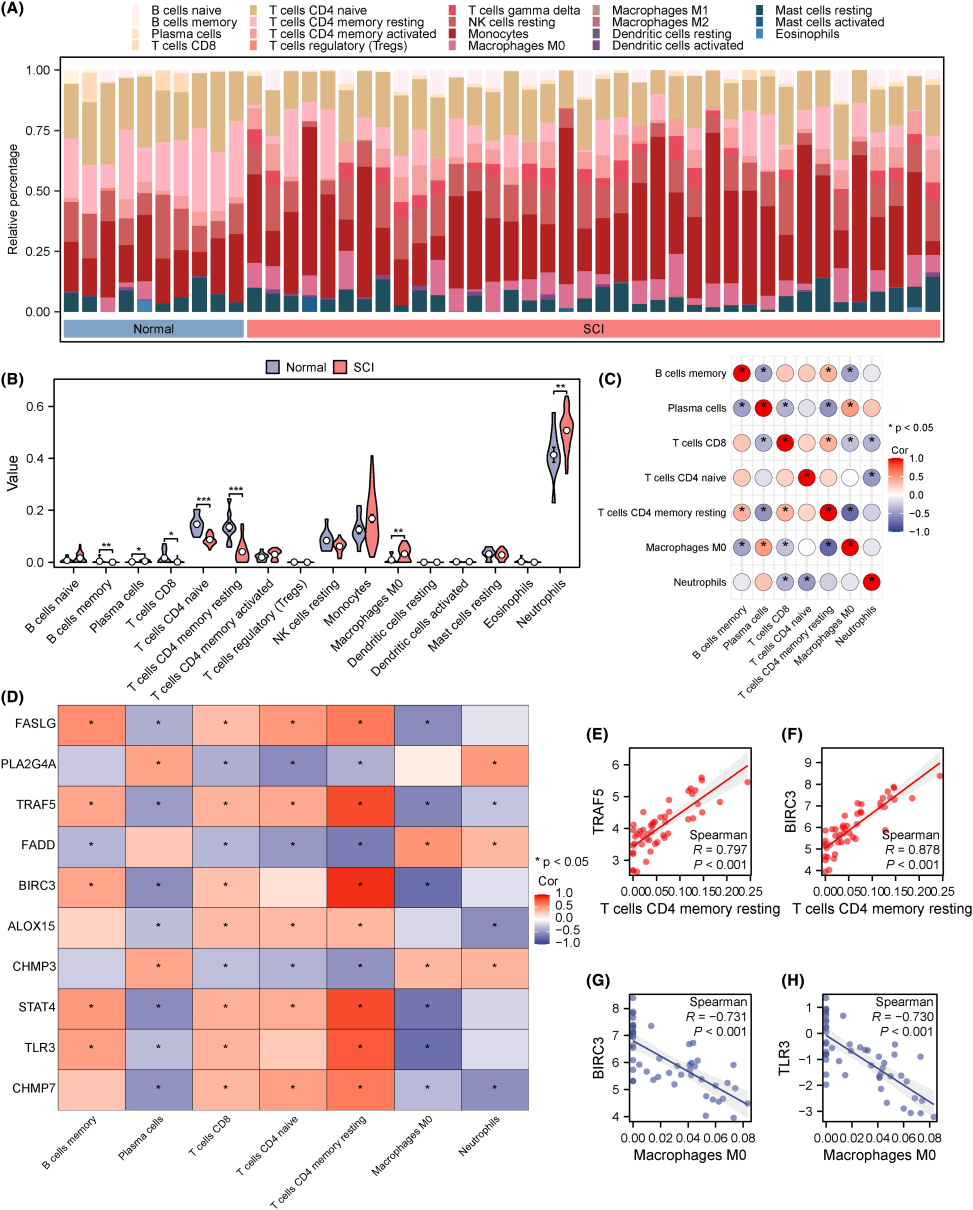
Immuno‐infiltration analysis of the GSE151371 dataset using CIBERSORTx. (A) Histogram depicting infiltrated immune cells in the GSE151371 dataset. (B) Group comparison plots of immune cell types in various subgroups of the GSE151371 dataset. (C) Heat map of correlations in various immune cell groups. (D) Heat map of correlations between immune cell types and hub genes. (E–H) Scatter plots of correlations between resting CD4^+^ memory T cells and *TRAF5* (E); resting CD4^+^ memory T cells and *BIRC3* (F); M0 macrophages and *BIRC3* (G); M0 macrophages and *TLR3* (H). *p < 0.05; **p < 0.01; ****p < 0.0001. ns, not significant.

Expression profile data in the GSE151371 dataset were compiled, followed by upload to the CIBERSORTx platform, which was utilized to analyse the associations of 22 infiltrated immune cell types in the SCI and normal groups. A histogram was plotted to highlight immune cell infiltration in the GSE151371 dataset (Figure 5A). In addition, group comparisons were carried out (Figure 5B) for the evaluation of variations in immune cell infiltration between the SCI and normal groups. The immune cells examined included memory B cells, naïve CD4^+^ T cells, resting CD4^+^ memory T cells, plasma cells, CD8^+^ T cells, M0 macrophages and neutrophils. These immune cells had marked differences in infiltration (*p* < 0.05). Additionally, a correlation heat map was generated for visualizing the associations of these seven groups of immune cells (Figure 5C) as well as of these cell types with hub genes (*BIRC3, FADD, TLR3, FASLG, TRAF5, ALOX15, CHMP3, PLA2G4A, CHMP7* and *STAT4*) (Figure 5D). The tightest positive correlations were obtained between resting CD4^+^ memory T cells, and *TRAF5* and *BIRC3*. In contrast, the most significant negative correlations were found between macrophages M0, and *BIRC3* and *TLR3*. These correlations were depicted using correlation scatter plots (Figure 5E–H).

### 3.7 Hub gene expression analysis

**FIGURE 6:**
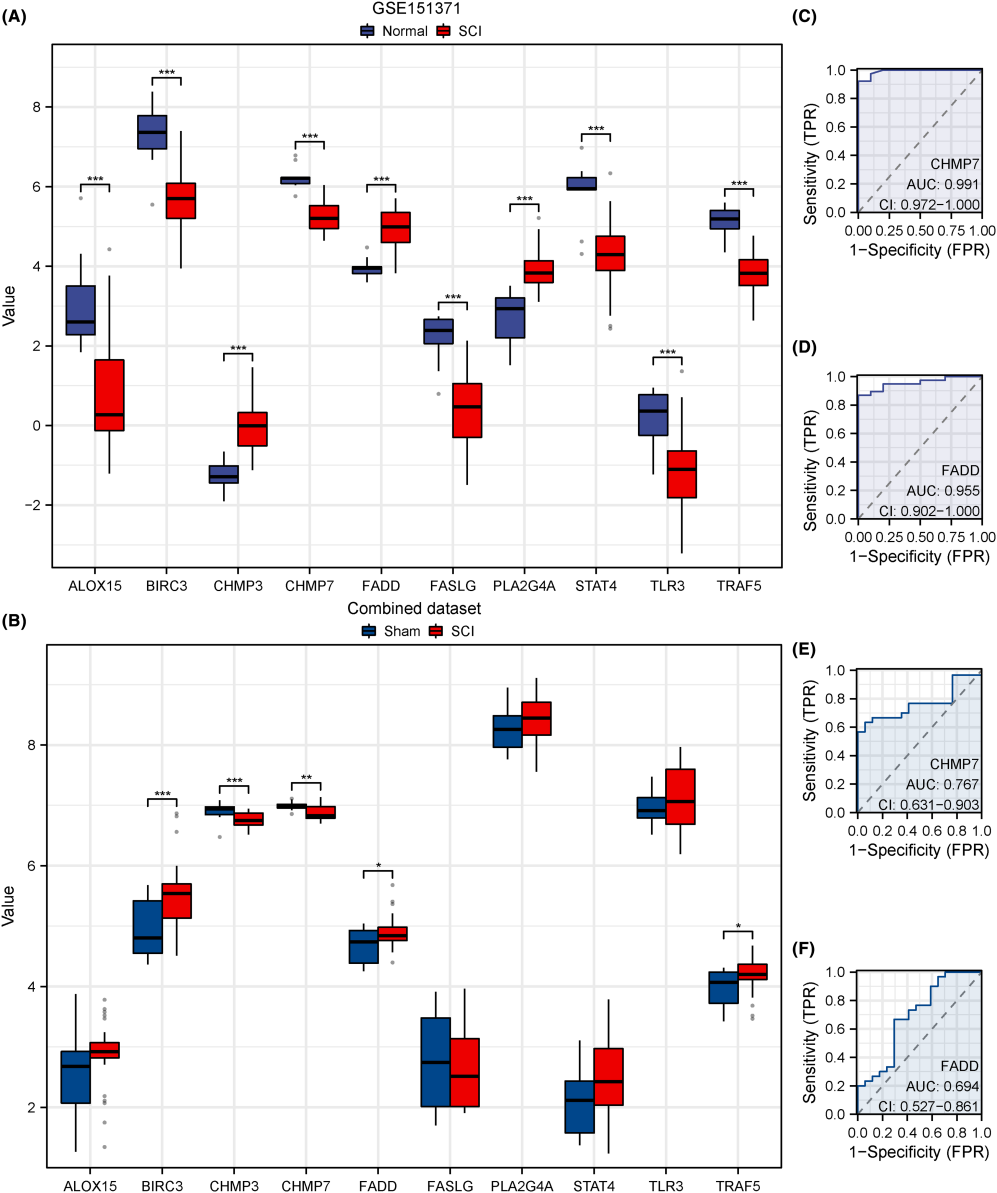
Expression analysis of hub genes. (A, B) Group comparison plots of hub genes in the GSE151371 (A) and combined (B) datasets. (C, D) ROC validation of *CHMP7* (C) and *FADD* (D) in the GSE151371 dataset. (E, F) ROC validation of *Chmp7* (E), and *Fadd* (F) in the combined dataset; the higher the AUC, the higher the diagnostic value. Low accuracy, AUC of 0.5–0.7; moderate accuracy, AUC of 0.7–0.9; high accuracy, AUC > 0.9. *p < 0.05; **p < 0.01; ****p < 0.0001. ns, not significant; ROC, receiver operating characteristic.

The rank‐sum test was conducted to detect variations in the levels of hub genes (*ALOX15, BIRC3, CHMP3, CHMP7, FASLG, FADD, PLA2G4A, STAT4, TRAF5* and *TLR3*) between the SCI and normal groups in GSE151371. The results were visualized using comparative plots (Figure 6A). Hub gene expression was validated using the combined mouse datasets GSE5296 and GSE47681 (Figure 6B). *CHMP7* and *FADD* exhibited significant (*p* < 0.05) expression differences and consistent expression trends. ROC curves were employed to illustrate the diagnostic values of *CHMP7* (Figure 6C) and *FADD* (Figure 6D) expression in SCI based on the GSE151371 dataset. The expression of *CHMP7* (AUC = 0.991, Figure 6C) and *FADD* (AUC = 0.955, Figure 6D) was highly accurate in the diagnosis of SCI. ROC curves were used to assess the diagnostic values of *Chmp7* (Figure 6E) and *Fadd* (Figure 6F) expression in SCI in the combined mouse dataset. *Chmp7* (AUC = 0.767, Figure 6E) and *Fadd* (AUC = 0.694, Figure 6F) expression had moderate accuracy in the diagnosis of SCI.

### 3.8 Validation of NRDEGs in the SCI model

**FIGURE 7:**
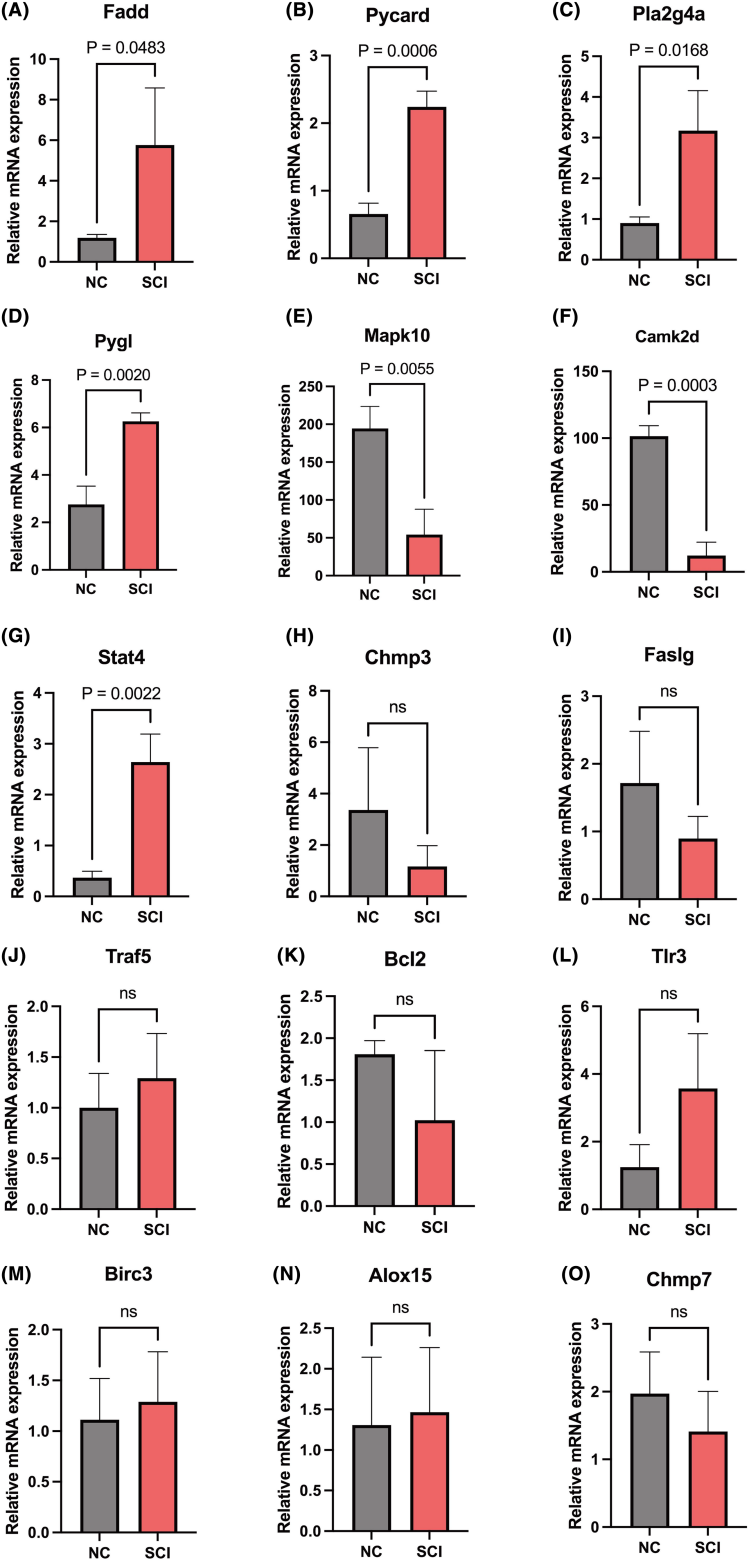
Expression of 15 NRDEGs measured in the spinal cord injury (SCI) and NC groups (*n* = 3/group). ns, not significant.

NRDEGs in the SCI model were assessed by qRT‐PCR to confirm the reliability of bioinformatics analyses. *Fadd, Pycard, Pla2g4a, Pygl* and *Stat4* were significantly upregulated in SCI mice (Figure 7A–D, G). Meanwhile, the expression levels of *Mapk10* and *Camk2d* were considerably lowered in the SCI model (Figure 7E,F). However, *Chmp3, Faslg, Traf5, Bcl2, Tlr3, Birc3, Alox15* and *Chmp7* had similar expression levels in the SCI and NC groups (Figure 7H–O).

## 4 DISCUSSION

The annual incidence of SCI, excluding deaths at the accident site, is 40 cases per million in the USA, corresponding to 12,000 new cases annually.^32^ However, current treatment options for SCI are inadequate. Wang et al.^33^ used the necrotic apoptosis inhibitor Nec‐1 to effectively mitigate necrotic apoptosis, reduce inflammation and ROS generation, and alleviate ischemic necrosis in the spinal cord following SCI. This research lays the groundwork for using necrotic apoptosis suppressors as a potential approach for treating spinal cord injuries. The association between inflammation and necroptosis following SCI is undeniable and significantly impacts patient prognosis. Therefore, the molecular targets and therapeutic mechanisms of SCI‐induced necroptosis were examined in this research. The current findings may help determine new therapeutic targets and drugs for SCI.

This study first explored NRDEGs in SCI using three datasets, i.e. GSE151371, GSE5296 and GSE47681. A total of 159 NRDEGs were obtained by merging them and removing duplicates from KEGG and MSigDB databases. Next, the intersection was determined between the DEGs and NRDEGs obtained from differential analysis of the SCI and normal groups in GSE151371. Finally, 15 DEGs associated with necroptosis after SCI were retrieved, and significant differences were observed in their expression. Among these, six genes were upregulated, and nine were downregulated.

The FADD belongs to the DD superfamily. These domains play a significant regulatory role in FAS‐ligand‐induced apoptotic signalling. Fas‐mediated cell death is important in CNS neurodegeneration.^34^, ^35^ For example, upregulation of Fas and FADD enhances cellular apoptosis following SCI.^36^, ^37^ Uni et al.^38^ found that the exogenous apoptotic pathway is induced by the interaction of TNF ligand with TNFR1, promoting the recruitment of TRADD, TRAF2, cIAP1/2 and RIPK1. Recruited FADD and caspase‐8 trigger caspase‐8 activation, which subsequently promotes caspase‐3/7 activation to enhance apoptotic cell death. However, the anti‐apoptotic protein cFLIP inhibits caspase‐8, whereas phosphorylation of RIPK1 and RIPK3 induces MLKL complex assembly, triggering necroptosis. This study confirmed the involvement of FADD in both necroptosis and apoptosis, suggesting that these processes are not mutually exclusive.

The phospholipase A2 (PLA2) superfamily has a significant role in SCI. PLA2 proteins catalyse the hydrolysis of glycerophospholipids in cell membranes, promoting the formation of free fatty acids and lysophospholipids. Arachidonic acid is a free fatty acid, whose metabolism involves two primary pathways, including the lipoxygenase pathway that generates leukotriene collections, and the cyclooxygenase pathway, which produces prostaglandins and thromboxanes.^39^ PLA2G4A, a rate‐limiting enzyme in arachidonic acid production, contributes to cellular inflammatory response by modulating the arachidonic acid metabolic pathway. Previous research has demonstrated that downregulation of PLA2G4A mitigates the adverse effects of ischemic brain injury.^40^ Lopez‐Vales et al.^41^ reported that SCI increased expression of cPLA2G4A, iPLA2G6A and sPLA2G2A of the PLA2 superfamily. cPLA2G4A plays a protective role after SCI, whereas, sPLA2G2A and iPLA2G6A contribute to secondary injury and functional impairment. This study provides strong evidence for PLA2G4A upregulation after SCI. SCI‐induced upregulation of cPLA2G4A exerts a protective effect and reduces necroptosis.

PYCARD (apoptosis‐associated speck‐like protein containing CARD, ASC) represents an important pro‐apoptotic protein composed of two structural domains, including the N‐terminal pyrin (PYD) and C‐terminal cysteine asparaginase (CARD) domains. The PYD domain allows PYCARD to bind to upstream signalling receptors, whereas the CARD domain enables the recruitment of activated effector proteins.^42^ PYCARD is an essential protein involved in the interactions between different molecules in the inflammatory complex. NLRP3 and CASP1 interact with PYCARD to assemble NLPR3 inflammatory vesicles, which contribute to the regulation of inflammation.^43^ PYCARD promotes CASP1 activation, leading to cellular inflammatory cascade response and apoptosis. Zhang and collaborators^44^ provided evidence suggesting that PYCARD potentially contributes to the pathogenesis of gouty arthritis. Significant positive correlations were noted between PYCARD mRNA and the proinflammatory cytokines IL‐6 and TNF‐α. Müller et al.^45^ found LCN2 induces inflammation following SCI by activating inflammatory vesicles and their constituents NLRP3, PYCARD, and CASP1. The maximum response was observed on the seventh day after SCI. Existing knowledge about the molecular mechanisms underlying necroptosis originates from studies of the TNF‐α‐induced necroptotic signalling pathway. TNF‐α, an inflammation‐associated cytokine, induces inflammatory responses, apoptosis and necroptosis under various pathophysiological conditions. In summary, SCI‐induced PYCARD expression contributes to the inflammatory response by inducing necroptosis. The present study identified a gap in the existing literature regarding mechanistic studies and bioinformatics analyses of the relationship between PYCARD expression and necroptosis after SCI, as revealed by a PubMed search. This study first reported the correlation between these parameters.

Necroptosis represents a regulated type of cell death involved in various diseases. ALOX15, BCL2, BIRC3, CAMK2D, FASLG, FADD, MAPK10, PLA2G4A, PYCARD, PYGL, STAT4, TRAF5 and TLR3 were significantly enriched in necroptosis and apoptosis pathways. Receptor‐interacting protein 3 (RIP3), belonging to the RIP family, represents a crucial modulator of the necroptotic pathway. According to current literature, RIP3 upregulates glycogen phosphorylase (PYGL) and glutamine synthase, which then triggers reactive oxygen species (ROS) production, thus promoting necroptosis.^46^, ^47^ Shang et al.^46^ hypothesized that reducing the C‐terminal expression of RIP3 may decrease MDA amounts and PYGL activity and potentially inhibit necroptosis, as determined by flow cytometry and LDH assays. Yu et al.^48^ showed that RIP3 phosphorylation activates RIP1 and promotes the activation of key glycolytic enzymes. Eventually, ROS accumulate and further promote necroptosis. The involvement of PYGL in the necroptosis pathway has been confirmed in previous studies, corroborating the current work.

The top 10 DEGs were selected as hub genes using PPI network analysis and the MCC algorithm. The current findings corroborate previous studies indicating *FASLG* expression is accompanied by the activation of FADD and caspases.^49^ It was hypothesized that necroptosis in SCI may share similarities with other mechanisms of cellular death. miRNAs represent small noncoding RNA molecules regulating gene expression, by affecting transcription or translation.^50^ A miRNA‐mRNA network was constructed to predict miRNAs that may regulate hub genes. Certain miRNAs that regulate multiple genes control the expression of hub genes. Chunquan et al.^51^ reported miR‐145‐5p upregulation and PLA2G4A downregulation in an astrocyte model of oxyglucose‐deprived astrocytes can effectively suppress cellular activity and inflammatory responses. Xiao et al.^52^ evaluated the effect of PLA2G4A on the transfection efficiency of a miRNA‐543 mimic in osteoarthritic (OA) chondrocytes. The work aimed to investigate the effects of targeting miRNA‐543 and PLA2G4A in OA chondrocytes. PLA2G4A expression was reduced in the mimic‐transfection and co‐transfection groups (miRNA‐543 mimic + PLA2G4A overexpressed plasmid) compared with the NC and PLA2G4A groups. In contrast, PLA2G4A expression was increased in the NC and mimic groups transfected with the PLA2G4A overexpression plasmid, compared with the co‐transfection group. Thus, PLA2G4A overexpression reversed the effects of the miRNA‐543 mimic in inducing cell viability and suppressing apoptotic and inflammatory pathways in OA chondrocytes. PLA2G4A had a negative correlation with miR‐543 and high expression levels in OA cartilage. The interaction between PLA2G4A and miR‐145‐5p/miR‐543 accorded with the findings of the above bioinformatics analysis. No previous reports on the interactions between hub genes and other miRNAs after SCI were found in a PubMed search, indicating the novelty of our findings.

Subsequently, correlations were assessed between the expression profiles of 22 immune cell types in the SCI and normal groups. The results highlighted that the expression of *TRAF5, BIRC3* and *TLR3* was closely associated with inflammation. The tightest positive correlations were observed between resting CD4^+^ memory T cells and *TRAF5* and *BIRC3*. The greatest negative correlations were observed between M0 macrophages and *BIRC3* and *TLR3*. Previous studies have reported a central role for macrophages in SCI progression.^53^ Therefore, this research suggests that *TRAF5, BIRC3* and *TLR3* could potentially induce the release of proinflammatory cytokines, thereby increasing immune cell infiltration.

However, this research had limitations. First, primary data for this study derived from online databases, and a small sample size was adopted. Second, qRT‐PCR was employed to verify the expression levels of all NRDEGs. *Camk2d, Fadd, Mapk10, Stat4, Pla2g4a, Pycard* and *Pygl* expression levels in the SCI model exhibited significant deviations (higher or lower) from those observed in the normal group. The identified hub genes should be validated in SCI samples through cell culture and animal experiments. Finally, because of the limited conditions, the current work lacked further mechanistic validation. Future research will address this limitation.

## 5 CONCLUSIONS

A total of 15 DEGs and 10 hub genes (*ALOX15, BIRC3, CHMP3, CHMP7, FASLG, FADD, PLA2G4A, STAT4, TRAF5* and *TLR3*) were found to be associated with SCI‐induced necroptosis. Additionally, shared DEGs in necroptosis and SCI were found. Our findings suggest that upregulation of *FADD* and downregulation of *CHMP7* following SCI might be indicative of necroptosis. Moreover, decreased expression of the *BIRC3* and *TLR3* genes might result in the infiltration of M0 macrophages, whereas, increased expression of *TRAF5* and *BIRC3* might enhance the infiltration of resting CD4^+^ memory T cells. In summary, the top 10 hub genes could potentially serve as diagnostic biomarkers for SCI‐induced necroptosis and provide a foundation and potential therapeutic target for SCI‐induced necrotizing apoptosis.

## AUTHOR CONTRIBUTIONS

**Jingcheng Liu:** Conceptualization (equal); investigation (equal); methodology (equal); software (equal); validation (equal); visualization (equal); writing – original draft (lead). **Jiang Cao:** Methodology (equal); software (lead); supervision (equal); validation (equal). **Xiao Yu:** Methodology (equal); software (equal); validation (equal). **Jie Chang:** Supervision (supporting). **Tao Sui:** Conceptualization (equal); methodology (equal); project administration (equal); supervision (equal); writing – review and editing (equal). **Xiaojian Cao:** Conceptualization (lead); funding acquisition (lead); project administration (lead); supervision (lead); writing – review and editing (lead).

## FUNDING INFORMATION

The current project was funded by the National Natural Science Foundation of China (#81871773, #82272484 and #82350002).

## CONFLICT OF INTEREST STATEMENT

The authors declare no conflict of interest.

## Supporting information


Figure S1:

Figure S2:

Figure S3:

Table S1:

Table S2:

Table S3:

Table S4:

Table S5:

Table S6:

Table S7:

Table S8:

Table S9:

## Data Availability

The data that support the findings of this study are openly available in GEO (GSE151371, GSE5296, and GSE47681).
