# Supplementary material for: Necroptosis pathway emerged as potential diagnosis markers in spinal cord injury
Source: J Cell Mol Med. 2024 Mar 20;28(7):e18219. doi: 10.1111/jcmm.18219 (PMC10955161; doi:10.1111/jcmm.18219)
Supplement: Supplementary file 1 — Figure S1: [file JCMM-28-e18219-s010.pdf]

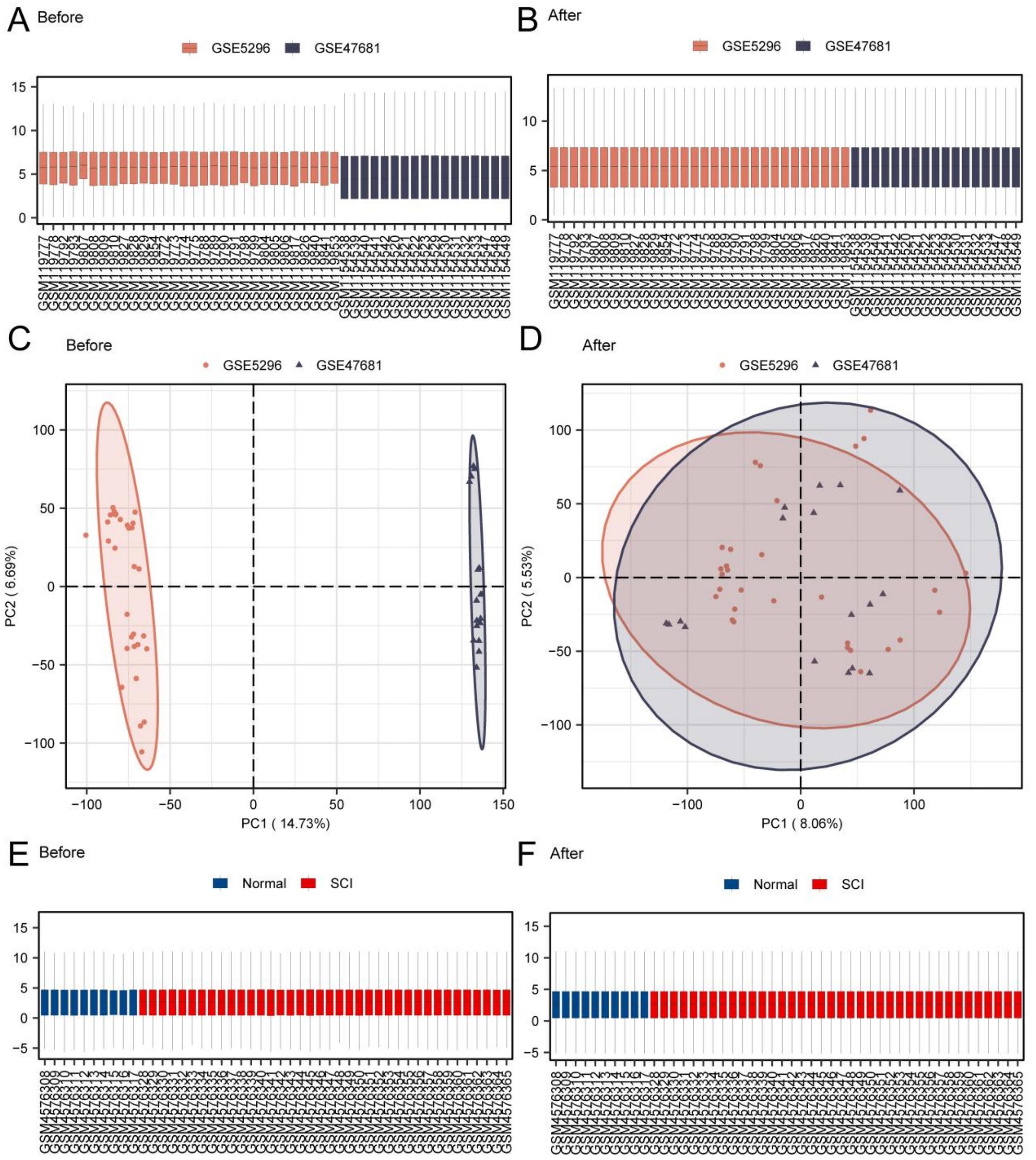

Fig. S1 Data Collection and Correction

Boxplots of the merged dataset prior to (A) and following (B) batch removal are shown. PCA plots of the merged dataset prior to (C) and following (D) batch removal are depicted. E. Boxplot of the GSE151371 dataset before correction. F. Boxplot of the GSE151371 dataset after correction.
