# Supplementary material for: Necroptosis pathway emerged as potential diagnosis markers in spinal cord injury
Source: J Cell Mol Med. 2024 Mar 20;28(7):e18219. doi: 10.1111/jcmm.18219 (PMC10955161; doi:10.1111/jcmm.18219)
Supplement: Supplementary file 2 — Figure S2: [file JCMM-28-e18219-s004.pdf]

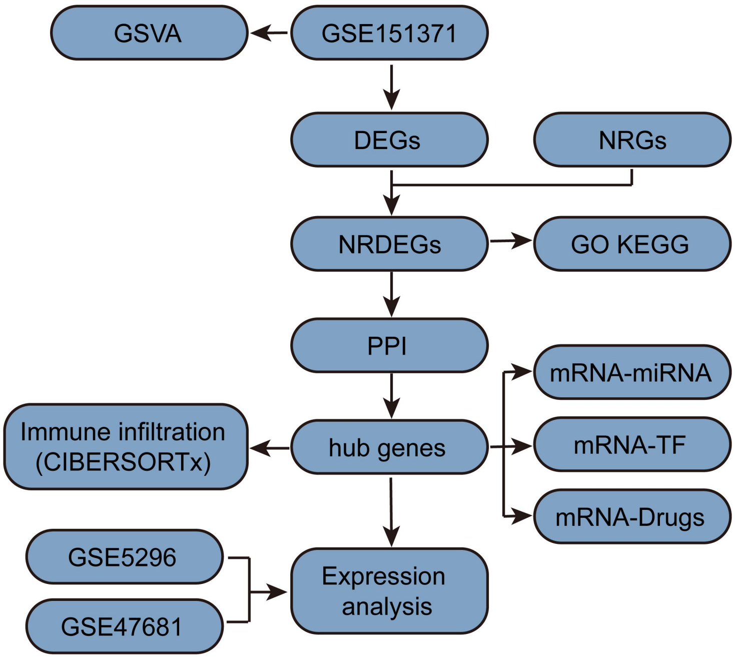

Fig. S2 Technological Roadmap

GSVA, Gene Set Variation Analysis; DEG, differentially expressed gene; NRG, necroptosis-related gene; NRDEG, necroptosis-related differentially expressed gene; GO, Gene Ontology; KEGG, Kyoto Encyclopedia of Genes and Genomes; PPI, protein-protein interaction; TF, transcription factor.
