# Supplementary material for: Necroptosis pathway emerged as potential diagnosis markers in spinal cord injury
Source: J Cell Mol Med. 2024 Mar 20;28(7):e18219. doi: 10.1111/jcmm.18219 (PMC10955161; doi:10.1111/jcmm.18219)
Supplement: Supplementary file 3 — Figure S3: [file JCMM-28-e18219-s008.pdf]

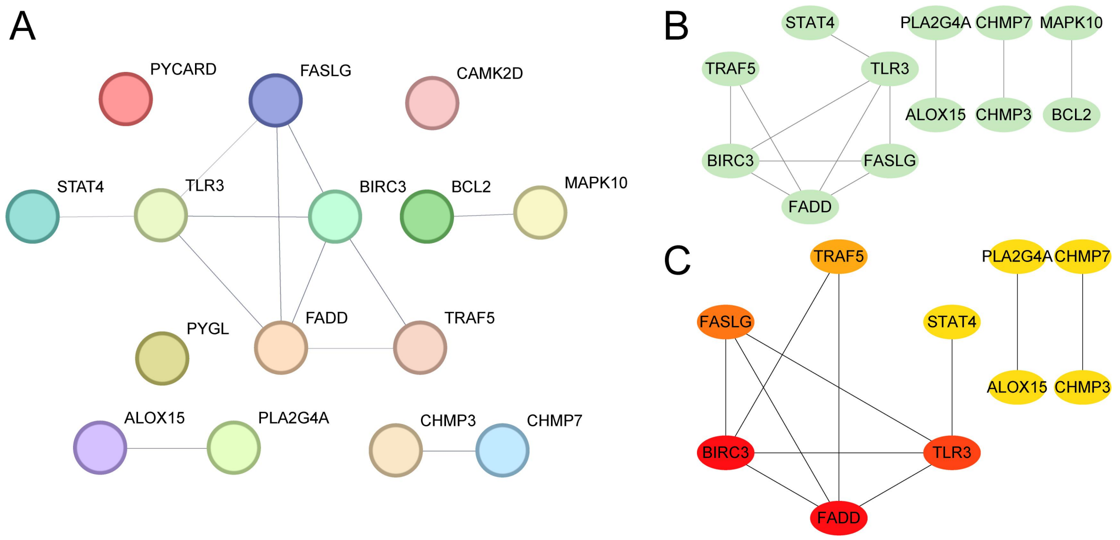

Fig. S3 Protein-protein interaction network

PPI networks based on STRING (A) and Cytoscape (B). C. PPI network of hub genes under the MCC algorithm; the color from light to dark represents scores from low to high.
